# Establishing key research questions for the implementation of artificial intelligence in colonoscopy: a modified Delphi method

**DOI:** 10.1055/a-1306-7590

**Published:** 2020-11-09

**Authors:** Omer F. Ahmad, Yuichi Mori, Masashi Misawa, Shin-ei Kudo, John T. Anderson, Jorge Bernal, Tyler M. Berzin, Raf Bisschops, Michael F. Byrne, Peng-Jen Chen, James E. East, Tom Eelbode, Daniel S. Elson, Suryakanth R. Gurudu, Aymeric Histace, William E. Karnes, Alessandro Repici, Rajvinder Singh, Pietro Valdastri, Michael B. Wallace, Pu Wang, Danail Stoyanov, Laurence B. Lovat

**Affiliations:** 1 Wellcome/EPSRC Centre for Interventional & Surgical Sciences, University College London, London, UK; 2 Digestive Disease Center, Showa University Northern Yokohama Hospital, Yokohama, Japan; 3 Clinical Effectiveness Research Group, Institute of Health and Society, University of Oslo, Oslo, Norway; 4 Department of Gastroenterology, Gloucestershire Hospitals NHS Foundation Trust, Gloucester, UK; 5 Computer Science Department, Universitat Autonoma de Barcelona and Computer Vision Center, Barcelona, Spain; 6 Center for Advanced Endoscopy, Beth Israel Deaconess Medical Center, Harvard Medical School, Boston, Massachusetts, USA; 7 Department of Gastroenterology and Hepatology, University Hospitals Leuven, TARGID KU Leuven, Leuven, Belgium; 8 Division of Gastroenterology, Vancouver General Hospital, University of British Columbia, Vancouver, British Columbia, Canada; 9 Division of Gastroenterology, Tri-Service General Hospital, National Defense Medical Center, Taipei, Taiwan; 10 Translational Gastroenterology Unit, John Radcliffe Hospital, Oxford, UK; 11 Oxford NIHR Biomedical Research Centre, University of Oxford, Oxford, UK; 12 Medical Imaging Research Center, ESAT/PSI, KU Leuven, Leuven, Belgium; 13 Hamlyn Centre for Robotic Surgery, Institute of Global Health Innovation, Imperial College London, London, UK; 14 Department of Surgery and Cancer, Imperial College London, London, UK; 15 Division of Gastroenterology and Hepatology, Mayo Clinic, Scottsdale, Arizona, USA; 16 ETIS, Universite de Cergy-Pointoise, ENSEA, CNRS, Cergy-Pointoise Cedex, France; 17 H. H. Chao Comprehensive Digestive Disease Center, Division of Gastroenterology & Hepatology, Department of Medicine, University of California, Irvine, California, USA; 18 Department of Gastroenterology, Humanitas Clinical and Research Center, IRCCS, Rozzano, Milan, Italy; 19 Humanitas University, Department of Biomedical Sciences, Pieve Emanuele, Milan, Italy; 20 Department of Gastroenterology and Hepatology, Lyell McEwan Hospital, Adelaide, South Australia, Australia; 21 School of Electronics and Electrical Engineering, University of Leeds, Leeds, UK; 22 Division of Gastroenterology & Hepatology, Mayo Clinic, Jacksonville, Florida, USA; 23 Department of Gastroenterology, Sichuan Academy of Medical Sciences and Sichuan Provincial People’s Hospital, Chengdu, China; 24 Gastrointestinal Services, University College London Hospital, London, UK

## Abstract

**Background** Artificial intelligence (AI) research in colonoscopy is progressing rapidly but widespread clinical implementation is not yet a reality. We aimed to identify the top implementation research priorities.

**Methods** An established modified Delphi approach for research priority setting was used. Fifteen international experts, including endoscopists and translational computer scientists/engineers, from nine countries participated in an online survey over 9 months. Questions related to AI implementation in colonoscopy were generated as a long-list in the first round, and then scored in two subsequent rounds to identify the top 10 research questions.

**Results** The top 10 ranked questions were categorized into five themes. Theme 1: clinical trial design/end points (4 questions), related to optimum trial designs for polyp detection and characterization, determining the optimal end points for evaluation of AI, and demonstrating impact on interval cancer rates. Theme 2: technological developments (3 questions), including improving detection of more challenging and advanced lesions, reduction of false-positive rates, and minimizing latency. Theme 3: clinical adoption/integration (1 question), concerning the effective combination of detection and characterization into one workflow. Theme 4: data access/annotation (1 question), concerning more efficient or automated data annotation methods to reduce the burden on human experts. Theme 5: regulatory approval (1 question), related to making regulatory approval processes more efficient.

**Conclusions** This is the first reported international research priority setting exercise for AI in colonoscopy. The study findings should be used as a framework to guide future research with key stakeholders to accelerate the clinical implementation of AI in endoscopy.

## Introduction

Artificial intelligence (AI)-based technologies are being developed at a rapid pace for gastrointestinal endoscopy, in particular for colonoscopy. Some AI-based systems have now achieved regulatory approval to assist colorectal polyp detection and characterization 1 . However, widespread clinical implementation in routine colonoscopy practice is not yet a reality.

The vast majority of AI research in endoscopy to date, and more broadly within healthcare, has focused on preclinical or retrospective studies. These studies have been crucial in the early phase of development 2 . However, following a number of a recent prospective randomized controlled trials (RCTs) for AI in colonoscopy, the emphasis is now quickly shifting along the translational roadmap to real-world implementation and clinical deployment 3 4 5 6 7 8 . Broad challenges related to the implementation of AI in medicine, including topics such as integration into existing clinical workflows, data sharing, transparency, and patient safety, have been highlighted by opinion and perspective articles 9 . Workshops have been conducted, for example by the National Institutes of Health and Radiological Society of North America, to identify key research priorities for AI in medical imaging, although this focused predominantly on foundational AI research topics, such as the development of new image reconstruction methods and novel machine-learning algorithms tailored to clinical imaging data 10 .

It is now widely recognized that the most translationally advanced AI applications in medicine, with the largest number of reported RCTs, belong to gastrointestinal endoscopy. The specialty is now in a prime position to become a leader for implementation research. Despite this, there has been minimal published literature exploring the opportunities and challenges relating to this critical next stage in endoscopy, which is likely to dominate the research agenda for the coming decade. In the field of colonoscopy, a recent review proposed some key principles for AI system development and testing 11 . However, to our knowledge, there has been no prior publication of a formal systematic process to identify research priorities for AI in endoscopy.

The aim of this study was to identify research priorities related to the implementation of AI in colonoscopy. Specific attention was given to colonoscopy, as AI systems are most translationally mature in this field of endoscopy. It was however expected that many of these AI implementation research priorities would be broadly applicable to general endoscopy.

## Methods

### Study design

A modified Delphi process is an established method for determining consensus opinion. This involves collating individual anonymized opinions from an expert group and establishing a consensus using an iterative process via a number of rounds. The research methodology used in this study was based on those previously published by the European Society of Gastrointestinal Endoscopy, European Association for Endoscopic Surgeons, and American Society of Colon and Rectal Surgeons to identify research priorities 12 13 14 . The Delphi methodology for research priority setting studies differs from the process typically used to create consensus statements, where a predefined threshold is determined for consensual agreement. Instead, for a research priority setting Delphi study, generated questions are scored through a number of rounds to finally establish a predefined number of top ranked questions.

For this study, the aim was to identify the top 10 ranked questions, in keeping with previously published major research priority setting studies 15 . A web-based, research electronic data capture (REDCap) survey was designed for the purposes of this Delphi process and was used in each round of the study. The study was conducted over a 9-month period between March 2019 and November 2019.

### Steering committee and expert participants

A steering committee consisting of translational endoscopists and computer scientists (O.F.A., L.B.L., J.T.A., P.V., D.S.E.) formed a key advisory group on the format and execution of this study.

The steering committee identified and invited participants by personal communication to create an international body of experts with experience in translational AI in colonoscopy. The following inclusion criteria were used: current involvement in clinician and engineer/computer scientist collaborative research in the field of AI or computer-aided diagnosis/detection (CAD) in colonoscopy with a specific focus on those with experience across the translation pipeline (i. e. case identification, data acquisition/curation, algorithm development, clinical evaluation, and deployment considerations). Publication history was also considered, with a requirement of at least one peer-reviewed publication in the field of AI/CAD in colonoscopy listed on PubMed. In addition, geographic diversity was considered to ensure representation from the major regions involved in AI research and development in endoscopy.

A total of 15 participants (12 endoscopists and three translational computer scientists/engineers) from nine countries were invited to form the expert group (see **Appendix 1s** , available in online-only Supplementary material); none declined to participate. The group included participants from North America (n = 5), Europe (n = 5), and the Asia–Pacific region (n = 5). The study aims and methodology were described from the outset of the study, with a clear primary objective to identify the top 10 research questions related to the implementation of AI in colonoscopy.

### Round 1: Question generation

All 15 participants were invited to list an unlimited number of research questions related to the implementation of AI and CAD in colonoscopy. The raw, verbatim responses were then collated to generate an anonymous long-list for review by the steering committee. Multiple responses addressing the same fundamental issue were consolidated into a single question, noting the number of times each issue was submitted. Responses were reviewed to ensure they were clearly understood. Care was taken to preserve the original meaning and avoid any amendment to the underlying theme where re-drafting was necessary. Responses that did not allow for the generation of clear research questions were excluded. The remaining responses were then categorized into nine broad themes for the purposes of round 2. Questions that could have been allocated to several categories were assigned to one by consensus amongst the steering committee.

### Round 2: Prioritization rating

Participants were asked to rank the questions generated from round 1 following the steering committee review, on a scale of 1 (low priority) to 5 (high priority). The questions were grouped into nine themes and presented in a randomized order. The survey software mandated that every question was assigned a score by each participant. The questions were ranked according to their total score. The steering committee reviewed the results and used the top 10 ranked questions including tied scores, which consisted of 28 questions in total, for re-ranking in round 3.

### Round 3: Final consensus rating

The top 10 ranked questions (including tied scores) generated from round 2 were redistributed to all participants for re-scoring. The questions were presented in rank order and with an associated mean score from round 2. On this occasion, participants were asked to re-score using a wider scale of 1 (very low priority) to 10 (very high priority) in order to gain greater discrimination between questions. The survey software mandated that every question was assigned a score by each participant. The final results were once again analyzed by the steering committee, using the total score, and in addition the percentage of very high priority responses (9 or 10 scores) for questions with tied rank, to identify the final top 10 research questions.

## Results

There was a complete (100 %) participant response rate for all three rounds of the study. The steering committee review and analysis of round 1 responses generated 59 individual research questions which are listed in **Appendix 2s** . These were categorized into nine themes as shown in Table 1 .

**Table 1:** The nine themes and numbers of questions generated for each.

Research theme	Number of questions
Data (access, sharing/privacy, curation)	8
Technological developments	11
Clinical adoption and integration into the endoscopy suite	10
Performance metrics, clinical trial design, and end points	10
Clinical applications	5
Training and education of workforce	3
Regulatory approval	3
Ethical and legal issues	6
Health economics	3

In round 2, the mean scores for the 59 questions, scored on a scale of 1 to 5, ranged from 2.69 to 4.63 ( **Table 1s** ). The top 10 ranked questions, including tied scores, that were redistributed for round 3 included a total of 28 questions from eight themes.

In round 3, the mean scores for the 28 questions, scored on a wider scale of 1 to 10, ranged from 6.13 to 8.80 ( Table 2 ). The percentage of responses scored as a very high priority (9 or 10) ranged from 7 % to 60 %.

**Table 2:** Questions in rank order following the final round 3 process.

Question	Rank	Total score	Mean score	Percentage of responses scored as very high priority (9 or 10)
What is the optimum clinical trial design to demonstrate efficacy for polyp detection AI/CAD software?	1	132	8.80	53
How do we improve the performance of AI/CAD to detect more challenging and advanced lesions (e. g. subtle flat lesions and sessile serrated lesions)?	2	126	8.40	47
How do we reduce false-positive rates for detection systems to avoid the user developing “alert fatigue”?	3	118	7.87	47
What are the optimal clinical end points for evaluation of AI/CAD?	4	118	7.87	27
Can we effectively combine polyp detection and characterization into one workflow?	5	116	7.73	60
Can we produce more efficient or automated annotation methods for data to reduce the burden on human experts?	6	115	7.67	40
How do we make the regulatory approval process more efficient and overcome hurdles?	7	113	7.53	33
How do we demonstrate that AI/CAD detection systems have an impact on interval colorectal cancer rates?	8	112	7.47	40
What is the optimum clinical trial design to demonstrate efficacy for polyp characterization (optical diagnosis) AI/CAD software?	9	112	7.47	27
How do we optimize CAD/AI so that it can be used in real-time with minimal latency?	10	111	7.40	53
What impact might AI/CAD detection and characterization systems have on colonoscopy surveillance intervals and what are the associated costs?	11	111	7.40	20
Can AI/CAD make endoscopy workflow more efficient (e. g. automated report writing)?	12	109	7.27	20
Can AI/CAD be used effectively to measure the quality of colonoscopy?	13	107	7.13	27
How should regulatory agencies deal with the iterative nature of software improvements in AI/CAD?	14	107	7.13	13
How do we develop quality assurance for annotation/labelling of data?	15	106	7.07	27
What impact will AI/CAD have on endoscopy training and performance?	16	105	7.00	33
How do we address data privacy, consent, and ownership issues to effectively share data across different countries and centers for AI/CAD development?	17	105	7.00	27
What effect will AI/CAD have on colonoscopy outcomes in relation to health economics (e. g. faster workflow, fewer colonoscopies, reduction in colorectal cancer rates) and how do we measure this?	18	105	7.00	20
How do we define standardized metrics for directly comparing the performance characteristics of different AI software?	19	104	6.93	13
How do we obtain enough data for categories that might be important for clinical application but are under-represented (e. g. dysplasia detection in inflammatory bowel disease)?	20	102	6.80	7
What performance thresholds (e. g. ASGE PIVI) are necessary to consider a resect & discard strategy when employing computer-aided diagnosis tools during colonoscopy?	21	100	6.67	27
Who owns the intellectual property in AI/CAD model development and can this be protected?	22	100	6.67	13
How do we audit AI/CAD systems once they are deployed in the clinical environment?	23	100	6.67	7
How do we train AI/CAD systems once they are deployed in order for them to improve and learn continuously in a clinical environment?	24	99	6.60	13
How do we develop large collaborative, standardized datasets for external validation of AI/CAD systems?	25	98	6.53	20
Could AI/CAD polyp detection and characterization systems distract endoscopists and impair performance?	26	97	6.47	13
How do we best train users/clinicians to critically evaluate the AI/CAD system including awareness of limitations to safeguard against incorrect AI/CAD decisions?	27	92	6.13	13
What is the best type of training data (videos, static images, or both) that should be used for developing polyp detection systems?	28	92	6.13	7

AI, artificial intelligence; CAD, computer-aided diagnosis/detection; ASGE, American Society for Gastrointestinal Endoscopy; PIVI, preservation and incorporation of valuable endoscopic innovations.

The final top 10 questions were from five themes: clinical trial design/end points (4 questions), technological developments (3 questions), clinical adoption/integration (1 question), data access/annotation (1 question), and regulatory approval (1 question). ( Table 3 ).

**Table 3:** Final top 10 questions grouped by themes.

Theme	Questions
Performance metrics, clinical trial design, and end points	What is the optimum clinical trial design to demonstrate efficacy for polyp detection AI/CAD software? What are the optimal clinical end points for evaluation of AI/CAD? How do we demonstrate that AI/CAD detection systems have an impact on interval colorectal cancer rates? What is the optimum clinical trial design to demonstrate efficacy for polyp characterization (optical diagnosis) AI/CAD software?
Technological developments	How do we improve the performance of AI/CAD to detect more challenging and advanced lesions (e. g. subtle flat lesions and sessile serrated lesions)? How do we reduce false-positive rates for detection systems to avoid the user developing “alert fatigue”? How do we optimize CAD/AI so that it can be used in real-time with minimal latency?
Clinical adoption and integration into endoscopy	Can we effectively combine polyp detection and characterization into one workflow?
Data (access, sharing/privacy, curation, and annotation)	Can we produce more efficient or automated annotation methods for data to reduce the burden on human experts?
Regulatory approval	How do we make the regulatory approval process more efficient and overcome hurdles?

AI, artificial intelligence; CAD, computer-aided diagnosis/detection.

## Discussion

This is the first international collaborative effort to systematically identify the research questions and priorities related to AI in colonoscopy with a particular focus on clinical implementation. In this study, an established modified Delphi method was used to determine the top 10 ranked research priorities, which were grouped into five broad themes.

The first theme, clinical trial design and related end points, predominates the list, containing four questions. The majority of published studies evaluating AI in colonoscopy are retrospective, evaluating algorithms outside the clinical environment, using datasets labelled by endoscopists. These studies often suffer from selection bias, for example by excluding cases that are challenging for AI or omitting low quality images. Moreover, these studies do not account for the real-world endoscopist – AI interaction. Ideally, AI technologies should be evaluated within the intended clinical pathway, reporting patient outcomes as end points 16 . For this reason, questions related to prospective evaluation and trial design rank highly in this study. However, retrospective in-silico studies, using carefully curated benchmark datasets, may be important for comparisons of different algorithms and for external validation purposes, particularly as they may allow for a more objective measure of standalone technical performance.

The top ranked questions include those related to optimum trial designs for polyp detection (CADe) and characterization (CADx). To date, among the published trials, there are only five RCTs for standalone CADe software, four parallel and one tandem in design 4 5 7 8 17 , and one prospective CADx trial 18 .

Many considerations regarding AI trial design are similar to the general evaluation of novel endoscopic technologies and have been discussed in detail elsewhere 19 . There are however unique challenges for AI trials. It can be difficult to account for the genuine contribution of AI assistance owing to potential operator bias and modification of endoscopist behavior. Some studies have used an independent observer, allowing for unblinding of AI outputs in missed-lesion scenarios, and one double-blind RCT deployed a sham AI system 5 . Such approaches can provide mechanistic insights; however, the definitions involved in these studies can be highly subjective. Another significant issue is that algorithm performance is also dependent on the quality of the procedure, which can be highly variable. Therefore, the selection of operators, for example low level or high level detectors, should be considered. Furthermore, performance errors can occur for AI models.

False-positive CADe outputs can be variably defined, often on the basis of duration or deemed clinical relevance, making direct comparisons between trials difficult. In one trial, the false-positive rate was not reported and instead the resection rate of non-neoplastic lesions was considered, which may be particularly relevant to device safety 8 .

CADx models can produce incorrect classifications or be designed to provide no output in cases of insufficient confidence. For CADx trials, evaluating the impact of AI on clinical workflow will depend upon its position within the clinical decision-making process: a second read, concurrent read, or independent diagnosis 20 21 . Special protocols for image acquisition, handling of poor-quality images, and additional time taken for analysis are important CADx considerations.

Another challenge for AI deployment is ensuring its generalizability to new clinical settings and populations. Ideally, external validation should occur, with models being evaluated in institutions where the training data were not collected.

Determining whether CADe systems have an impact on interval colorectal cancer (CRC) would likely require long-term longitudinal follow-up and reliable linkage to cancer registries. Given that post-colonoscopy cancer is a relatively rare outcome, long-term studies would need to be large and well designed to account for the potential confounders. Ideally long-term outcomes for patients randomized to AI assistance or standard colonoscopy would provide some insight; however, the associated financial costs of designing an adequately powered and robust study may be a barrier.

To advance the first theme, dedicated AI endoscopy working groups, ideally created by professional societies, should aim to consolidate trial designs and produce robustly defined outcome measures. Recently, an international working group produced the CONSORT-AI and SPIRIT-AI extensions, aimed specifically at promoting standardized and transparent reporting of AI interventional trials 16 . Our study could be used to address the additional challenges specific to AI in colonoscopy and develop recommendations for the design and reporting of AI trials in endoscopy.

The second theme, technological developments, includes three questions. The first relates to how we can improve CADe systems to detect more challenging and advanced lesions. To date, the published CADe RCTs have demonstrated a significant increase only in the detection of non-advanced adenomas, as summarized by a recently published meta-analysis 22 . It has long been debated whether the additional detection of non-advanced lesions actually translates into any reduction in interval CRC. It is not unreasonable therefore to focus development of CADe systems to detect advanced lesions, particularly challenging lesions that may otherwise be overlooked.

There have been very limited preclinical studies assessing the ability of CADe to detect sessile serrated lesions (SSLs) and advanced flat lesions, such as laterally spreading tumors (LSTs). A recent review evaluated the training and test datasets for CADe studies with at least 100 lesions, demonstrating that the majority of studies did not differentiate across the type of flat lesions, especially for non-granular LSTs, most likely due to their low population prevalence 23 . Furthermore, retrospective studies and endoscopic datasets may suffer from a selection bias, containing optimally captured images. Future research should focus on creating enriched datasets with images of subtle advanced flat lesions and SSLs, particularly in scenarios where human perceptual errors can occur. Moreover, prospective trials in higher risk patient populations may actually allow us to determine if the use of AI translates to increased detection of these subtle lesions.

The second question within this theme asks how we could potentially reduce the false-positive rates associated with CADe. False-positive outputs could be problematic by leading to “user fatigue.” To date, prospective trials have not suggested that false-positive outputs have significantly impacted on workflow. Nevertheless, it would be advantageous to reduce false-positive outputs. Retraining of algorithms with scenarios that currently lead to false-positives could be a simple mechanism, whilst other approaches may include the use of recurrent neural networks, which have memory and can process temporal sequences of frames, mimicking the behavior of human endoscopists. Further research on the acceptable false-positive rate for endoscopists may be useful but also it should be recognized that CADe systems are currently designed as “red flag” techniques. Dedicated “challenges” or competitions, co-developed between computer scientists and endoscopists, aimed at tackling a specific problem have proved beneficial in the past 24 . Such a challenge aimed at addressing false-positives could be invaluable in helping to identify state of the art approaches.

The third question within the technical development theme highlights the challenge of latency, which refers to the delay between the display of an endoscopic image frame and the output from the AI system. Minimal latency is crucial, particularly for CADe systems, where real-time highlighting of lesions is required. The degree of latency could also be a limitation when AI is deployed using cloud- or server-based computing. There are published studies evaluating acceptable levels of latency for telesurgery 25 . Further similar research is required to specifically identify acceptable latency levels for endoscopy.

The theme of clinical adoption and integration into the endoscopy includes one question, which concerns the effective combination of polyp detection and characterization into one workflow. This approach could mitigate the effect of increased detection by CADe systems of diminutive hyperplastic polyps, particularly in the rectosigmoid, potentially avoiding unnecessary polypectomies 26 . However, the design of such a system, particularly with seamless transition from detection to characterization of the same lesion, may be challenging. To date, no prospective study has been published that evaluates a system combining both CADe and CADx into one workflow, although demonstrations have been published as a video case report and abstract 27 28 . Future research should specifically address workflow challenges, such as the ability to reliably detect and characterize the same unique polyp when switching from white light to virtual chromoendoscopy, dealing with instances when multiple polyps are in view, and preferably avoiding the need for manual selection of a region of interest. Additional CADx studies that use only white light to predict histopathology would also be valuable as highlighted by a recent preliminary study 29 .

There is one question in the data theme that relates to developing more efficient or automated annotation methods to reduce the burden on human experts. Currently, most AI algorithms are developed using a fully supervised learning approach. This requires manual annotation of large numbers of endoscopic image frames. This can be incredibly time-consuming and expensive, particularly in medical applications where domain expertise is required. Automated or semi-automated annotation strategies based on machine learning approaches that mimic human annotators are promising areas for future research 30 . Moreover, research aimed at active learning approaches, where algorithms iteratively determine which unlabeled data samples should be annotated by the human could dramatically improve efficiency. Dedicated computer vision competitions or “challenges” for endoscopic video labelling could help accelerate progress in this area further. The creation of datasets for this purpose requires careful co-development between endoscopists and computer scientists. A recent publication provided an overview of existing endoscopic datasets available for AI research, highlighting that few exist and the majority are relatively small 31 .

The final theme and question in our top 10 priorities concerns improving the efficiency of the regulatory approval process. It is generally accepted that AI-based technologies can differ from traditional software as a medical device (SaMD). A recent review article provided an overview of regulatory pathways in relation to gastrointestinal endoscopy 32 . Current regulatory approval pathways for AI are evolving and the associated uncertainty could delay clinical translation. Regulatory pathways differ globally, although the International Medical Device Regulators Forum is a voluntary group that develops harmonized principles for SaMD. Clearly a balance must be achieved between promoting innovation and ensuring patient safety. It is possible that greater collaboration between regulators and other stakeholders, including AI developers and clinicians, may lead to more streamlined pathways for clinical translation.

It is noteworthy that the 11th ranked question, with an equal mean score to the 10th ranked question but a lower proportion of very high priority scores, belongs to the healthcare economics theme, which is likely to be crucial for widespread implementation. The impact of CADe and CADx systems on colonoscopy surveillance intervals and associated financial costs warrants further investigation, as it will likely underpin reimbursement policies. One study has just been published, as an add-on to a previous CADx clinical trial, which demonstrated that AI assistance specifically for a diagnose-and-leave strategy resulted in significant cost reductions for colonoscopy when considering public health insurance systems in four countries 33 .

There are several limitations to our study. Although we used methods based on previously published research priority setting exercises, bias can be introduced at different stages of the Delphi process. Questions were consolidated and reformatted by the steering committee, which could lead to inadvertent changes to the underlying theme; however, such changes were only made where absolutely necessary and efforts were made to preserve the original meaning.

Another limitation relates to the sample size of experts: although it was acceptable for a Delphi study, the group was relatively small owing to the specific selection of translational researchers currently involved in AI implementation. This was mitigated to some extent by allowing an unlimited number of questions to be generated, leading to a comprehensive and thematically diverse long-list. Furthermore, whilst there was clear discrimination of the very top priorities, the remaining question scores were narrowly distributed, possibly because of the small sample size. Owing to rapid growth in the field, our findings could now be validated with a larger group of translational AI researchers. The creation of a database of translational AI researchers, perhaps by dedicated working groups within professional societies, would assist validation, reduce potential selection bias, and also benefit future collaborative research in the field.

It is also important to emphasize that the top 10 priorities were identified by an expert group who are involved in translational research and focused on advancing clinical implementation at this point in time. The priorities include likely short-term barriers, largely related to AI evaluation and technical issues, that could soon be addressed. Therefore, repeating the exercise in 5 years’ time would be valuable. Furthermore, inclusion of a wider range of stakeholders, including endoscopists not involved in AI development, patients, public health researchers, and ethicists, could have resulted in a different ranking of top priorities.

In conclusion, this is the first reported international research priority setting exercise for AI in colonoscopy. Although specific attention was given to colonoscopy, the majority of the themes and key research questions will apply to the use of AI in general endoscopic practice. The results from this study provide a comprehensive framework to stimulate further discussions and collaborative research amongst the key stakeholders involved in AI implementation, with a view to accelerating the translation of effective AI systems in endoscopy.

## Appendix 1s, 2s, Table 1s

Supplementary material Zusatzmaterial
