# Supplementary material for: Establishing key research questions for the implementation of artificial intelligence in colonoscopy: a modified Delphi method
Source: Endoscopy. 2020 Nov 9;53(9):893–901. doi: 10.1055/a-1306-7590 (PMC8390295; doi:10.1055/a-1306-7590)
Supplement: Supplementary file 1 — Supplementary material [file 19561supmat_10-1055-a-1306-7590.pdf]

Supplementary material

Appendix 1s: Participants in the Delphi group

Jorge Bernal, Tyler M. Berzin, Raf Bisschops, Michael F. Byrne, Peng-Jen Chen, James E. East, Suryakanth R. Gurudu, Aymeric Histace, William E. Karnes, Masashi Misawa, Yuichi Mori, Rajvinder Singh, Danail Stoyanov, Michael B. Wallace, Pu Wang.

Appendix 2s: Questions generated from Round 1 and categorized by the steering committee

Parentheses indicate the number of times that multiple responses addressing the same fundamental issue were consolidated into a single question.

Data (access, sharing/privacy, curation and annotation)

1. Can we produce more efficient or automated annotation methods for data to reduce the burden on human experts?

2. How do we address data privacy, consent and ownership issues to effectively share data across different countries and centers for AI/CAD development? (4)

3. Once a CAD/AI system is developed, should the system be additionally trained by input data from the purchasing centers and is it acceptable for these data to be shared with the software developer?

4. How do we develop quality assurance for annotation/labelling of data?

5. How do we improve the quality of video capture and recording for AI/CAD development?

6. How do we develop large collaborative, standardised datasets for external validation of AI/CAD systems? (3)

7. Who owns the intellectual property in AI/CAD model development and can this be protected?

8. How do we obtain enough data for categories that might be important for clinical application but are under-represented? e.g. dysplasia detection in inflammatory bowel disease

Technological Developments

9. Which type of computer vision technique is optimal for polyp detection and characterisation, is deep-learning the best approach?

Supplementary material

10. What is the optimum number of images needed for effective CAD/AI development for polyp detection and characterisation?

11. What is the best type of training data (videos, static images or both) that should be used for developing polyp detection systems? (3)

12. How do we optimise CAD/AI so that it can be used in real-time with minimal latency?

13. How do we most effectively create an environment for knowledge exchange to overcome AI/CAD translational challenges and discuss recent advances? E.g. 'challenges or competitions' organised by computer scientists and clinicians

14. How do we train AI/CAD systems once they are deployed in order for them to improve and learn continuously in a clinical environment?
15. How do we improve the performance of AI/CAD to detect more challenging and advanced lesions? (e.g. subtle, flat lesions and sessile serrated lesions?) (3)

16. Can we use synthetic or computer-generated images for training and evaluation of AI/CAD systems? (2)

17. Can we develop one AI/CAD system that is independent to a specific type of manufacturer i.e. works for all endoscope processors?

18. How do we reduce false positive rates for detection systems to avoid the user developing 'alert fatigue'?

19. Do AI/CAD algorithms vary in performance due to geographical or genetic changes in the underlying data?

Clinical adoption and Integration into endoscopy suite

20. Which are the best user interface designs and alert mechanisms e.g. visual or other, that can be used in the endoscopy suite for a detection system? (3)

21. Can we effectively combine polyp detection and characterisation into one workflow?

22. Does the most effective user interface design vary according to patient factors and quality of colonoscopy performed?

23. Should we use one integrated monitor for AI/CAD or a separate second monitor to display outputs? (3)

24. Could AI/CAD polyp detection and characterisation systems distract endoscopists and impair performance?

Supplementary material

25. Can AI/CAD make endoscopy workflow more efficient? e.g. automated report writing

26. What is the ideal role of CAD/AI systems in decision making particularly for polyp characterisation e.g. decision support (first reader, second reader) or independent stand-alone reader? (3)

27. Do we need to develop ‘explainable AI’ models for clinical adoption and user acceptance?

28. What are the main barriers for widespread clinical adoption for AI/CAD software in colonoscopy?

29. What are the technical requirements for deploying AI/CAD systems into the endoscopy suite and what are the implications? e.g. installation of GPUs, cloud-based systems (3)

Performance metrics, clinical trial design and endpoints

30. What is the optimum clinical trial design to demonstrate efficacy for polyp detection AI/CAD software?

31. What is the optimum clinical trial design to demonstrate efficacy for polyp characterisation (optical diagnosis) software? (5)

32. How do we demonstrate that AI/CAD detection systems have an impact on interval colorectal cancer rates? (2)

33. What performance thresholds (e.g. ASGE PIVI) are necessary to consider a resect & discard strategy when employing computer-aided diagnosis tools during colonoscopy? (3)

34. Should AI/CAD always be validated in an independent centre which was not involved in algorithm development to avoid bias and conflict of interest?

35. How can we account for observer bias in AI/CAD trials in colonoscopy?

36. How do we define standardised metrics for directly comparing the performance characteristics of different AI software? (6)

37. How do we overcome issues of defining a gold standard for pathology of sessile serrated polyps/lesions so that we can train AI models for optical diagnosis?

38. How do we overcome the selection bias that is often present in retrospective studies using AI/CAD?

Supplementary material

39. What are the optimal clinical endpoints for evaluation of AI/CAD? (3)

Clinical Applications

40. Can AI/CAD be used effectively to automatically detect anatomical landmarks in clinical practice?

41. Can we develop a polyp characterisation system to provide therapeutic decision-making recommendations based on depth of invasion? E.g. piecemeal EMR, ESD, Surgery

42. Can AI/CAD be used effectively to measure the quality of colonoscopy? (2)

43. Can AI/CAD be used to obtain more objective measurements of polyp size?

44. Can AI/CAD be used effectively to automatically score and report bowel preparation in clinical practice?

Training and Education of Workforce

45. Which type of endoscopists (e.g. trainees, non-experts or experts) benefit most from using AI/CAD software for polyp detection and characterisation during colonoscopy?

46. What impact will AI/CAD have on endoscopy training and performance? (3)

47. How do we best train users/clinicians to critically evaluate the AI/CAD system including awareness of limitations to safeguard against incorrect AI/CAD decisions? (2)

Regulatory Approvals

48. How do we make the regulatory approval process more efficient and overcome hurdles? (4)

49. How should regulatory agencies deal with the iterative nature of software improvements in AI/CAD?

50. How do we audit AI/CAD systems once they are deployed in the clinical environment?

Supplementary material

Ethical and Legal Issues

51. If AI/CAD can measure the quality of colonoscopy e.g. post-hoc analysis for missed polyps or other performance characteristics, what are the implications of this? (2)

52. How do we best learn from other specialities who have used AI/CAD in clinical practice e.g. radiology, to understand potential pitfalls?

53. If clinical harm occurs due to a CAD/AI related error, who is accountable? (2)

54. If AI/CAD becomes commonplace, would endoscopists who do not use AI to enhance their practice be liable for errors e.g. missed cancers?

55. Could the increase in detection of diminutive adenomas and clinically irrelevant lesions using AI/CAD detection systems actually be harmful by drawing focus away from actions that might find more hidden advanced lesions?

56. Do we need to store electronic images and data from all AI/CAD predictions used in clinical practice? e.g. for post-colonoscopy colorectal cancer case reviews

Health Economics

57. What effect will AI/CAD have on colonoscopy outcomes in relation to health economics and how do we measure this? E.g. faster workflow, fewer colonoscopies, reduction in colorectal cancer rates (4)

58. If AI/CAD technology is expensive could it lead to increased inequality between different healthcare providers?

59. What impact might AI/CAD detection and characterisation systems have on colonoscopy surveillance intervals and what are the associated costs?

Supplementary material

Table 1s Ranking of all 59 questions following Round 2 process

Question	Rank (= indicates tied)	Mean score (scale 1-5)
What is the optimum clinical trial design to demonstrate efficacy for polyp detection AI/CAD software?	1	4.63
How do we improve the performance of AI/CAD to detect more challenging and advanced lesions? (e.g. subtle, flat lesions and sessile serrated lesions?)	2	4.44
Can we produce more efficient or automated annotation methods for data to reduce the burden on human experts?	=3	4.38
How do we optimise CAD/AI so that it can be used in real-time with minimal latency?	=3	4.38
How do we reduce false positive rates for detection systems to avoid the user developing 'alert fatigue'?	4	4.25
Who owns the intellectual property in AI/CAD model development and can this be protected?	=5	4.13
Can we effectively combine polyp detection and characterisation into one workflow?	=5	4.13
What is the optimum clinical trial design to demonstrate efficacy for polyp characterisation (optical diagnosis) AI/CAD software?	=5	4.13
What are the optimal clinical endpoints for evaluation of AI/CAD?	=5	4.13
How do we develop quality assurance for annotation/labelling of data?	6	4.06
How do we address data privacy, consent and ownership issues to effectively share data across different countries and centers for AI/CAD development?	=7	4.00
What is the best type of training data (videos, static images or both) that should be used for developing polyp detection systems?	=7	4.00
How do we train AI/CAD systems once they are deployed in order for them to improve and learn continuously in a clinical environment?	=7	4.00

Supplementary material

How do we demonstrate that AI/CAD detection systems have an impact on interval colorectal cancer rates?	=7	4.00
What performance thresholds (e.g. ASGE PIVI) are necessary to consider a resect & discard strategy when employing computer-aided diagnosis tools during colonoscopy?	=7	4.00
How do we make the regulatory approval process more efficient and overcome hurdles?	=7	4.00
Can AI/CAD make endoscopy workflow more efficient? e.g. automated report writing	=8	3.94
What impact will AI/CAD have on endoscopy training and performance?	=8	3.94
How do we develop large collaborative, standardised datasets for external validation of AI/CAD systems?	=9	3.88
How do we obtain enough data for categories that might be important for clinical application but are under-represented? e.g. dysplasia detection in inflammatory bowel disease	=9	3.88
How do we define standardised metrics for directly comparing the performance characteristics of different AI software?	=9	3.88
How do we audit AI/CAD systems once they are deployed in the clinical environment?	=9	3.88
What effect will AI/CAD have on colonoscopy outcomes in relation to health economics and how do we measure this? E.g. faster workflow, fewer colonoscopies, reduction in colorectal cancer rates	=9	3.88
What effect will AI/CAD have on colonoscopy outcomes in relation to health economics and how do we measure this? E.g. faster workflow, fewer colonoscopies, reduction in colorectal cancer rates	=9	3.88
Could AI/CAD polyp detection and characterisation systems distract endoscopists and impair performance?	=10	3.81
Can AI/CAD be used effectively to measure the quality of colonoscopy?	=10	3.81

Supplementary material

How do we best train users/clinicians to critically evaluate the AI/CAD system including awareness of limitations to safeguard against incorrect AI/CAD decisions?	=10	3.81
How should regulatory agencies deal with the iterative nature of software improvements in AI/CAD?	=10	3.81
Can we develop one AI/CAD system that is independent to a specific type of manufacturer i.e. works for all endoscope processors?	=11	3.75
Which are the best user interface designs and alert mechanisms e.g. visual or other, that can be used in the endoscopy suite for a detection system?	=11	3.75
Should we use one integrated monitor for AI/CAD or a separate second monitor to display outputs?	=11	3.75
How do we overcome the selection bias that is often present in retrospective studies using AI/CAD?	=11	3.75
Can we develop a polyp characterisation system to provide therapeutic decision-making recommendations based on depth of invasion? E.g. piecemeal EMR, ESD, Surgery	=11	3.75
Should AI/CAD always be validated in an independent centre which was not involved in algorithm development to avoid bias and conflict of interest?	=12	3.69
How do we overcome issues of defining a gold standard for pathology of sessile serrated polyps/lesions so that we can train AI models for optical diagnosis?	=12	3.69
How do we most effectively create an environment for knowledge exchange to overcome AI/CAD translational challenges and discuss recent advances? E.g. 'challenges or competitions' organised by computer scientists and clinicians	=13	3.63
If clinical harm occurs due to a CAD/AI related error, who is accountable?	=13	3.63
How do we improve the quality of video capture and recording for AI/CAD development?	=14	3.56
How can we account for observer bias in	=14	3.56

Supplementary material

AI/CAD trials in colonoscopy?		
Can AI/CAD be used to obtain more objective measurements of polyp size?	=14	3.56
Which type of endoscopists (e.g. trainees, non-experts or experts) benefit most from using AI/CAD software for polyp detection and characterisation during colonoscopy?	=14	3.56
Once a CAD/AI system is developed, should the system be additionally trained by input data from the purchasing centers and is it acceptable for these data to be shared with the software developer?	=15	3.50
Which type of computer vision technique is optimal for polyp detection and characterisation, is deep-learning the best approach?	=15	3.50
What is the optimum number of images needed for effective CAD/AI development for polyp detection and characterisation?	=15	3.50
What are the technical requirements for deploying AI/CAD systems into the endoscopy suite and what are the implications? e.g. installation of GPUs, cloud-based systems	=15	3.50
If AI/CAD technology is expensive could it lead to increased inequality between different healthcare providers?	=15	3.50
Can AI/CAD be used effectively to automatically score and report bowel preparation in clinical practice?	=16	3.44
Could the increase in detection of diminutive adenomas and clinically irrelevant lesions using AI/CAD detection systems actually be harmful by drawing focus away from actions that might find more hidden advanced lesions?	=16	3.44
Do we need to store electronic images and data from all AI/CAD predictions used in clinical practice? e.g. for post-colonoscopy colorectal cancer case reviews	=16	3.44
What is the ideal role of CAD/AI systems in decision making particularly for polyp characterisation e.g. decision support (first reader, second reader) or independent stand-alone reader?	=17	3.31
What are the main barriers for widespread clinical adoption of AI/CAD software in	=17	3.31

Supplementary material

colonoscopy?		
If AI/CAD becomes commonplace, would endoscopists who do not use AI to enhance their practice be liable for errors e.g. missed cancers?	=17	3.31
How do we best learn from other specialities who have used AI/CAD in clinical practice e.g. radiology, to understand potential pitfalls?	18	3.25
If AI/CAD can measure the quality of colonoscopy e.g. post-hoc analysis for missed polyps or other performance characteristics, what are the implications of this?	19	3.19
Can AI/CAD be used effectively to automatically detect anatomical landmarks in clinical practice?	20	3.13
Do AI/CAD algorithms vary in performance due to geographical or genetic changes in the underlying data?	21	3.00
Can we use synthetic or computer-generated images for training and evaluation of AI/CAD systems?	=22	2.88
Does the most effective user interface design vary according to patient factors and quality of colonoscopy performed?	=22	2.88
Do we need to develop 'explainable AI' models for clinical adoption and user acceptance?	23	2.69
